# Neuroactive Peptides as Putative Mediators of Antiepileptic Ketogenic Diets

**DOI:** 10.3389/fneur.2014.00063

**Published:** 2014-04-29

**Authors:** Carmela Giordano, Maddalena Marchiò, Elena Timofeeva, Giuseppe Biagini

**Affiliations:** ^1^Laboratory of Experimental Epileptology, Department of Biomedical, Metabolic and Neural Sciences, University of Modena and Reggio Emilia, Modena, Italy; ^2^Neuropediatric Unit, Department of Medical and Surgical Sciences for Children and Adults, Policlinico Hospital, University of Modena and Reggio Emilia, Modena, Italy; ^3^Department of Neurosciences, NOCSAE Hospital, Modena, Italy; ^4^Département Psychiatrie et Neurosciences, Faculté de Médecine, Centre de Recherche de l’Institut Universitaire de Cardiologie et de Pneumologie de Québec, Université Laval, Québec, QC, Canada

**Keywords:** adiponectin, fasting, ghrelin, ketogenic diet, neuropeptide Y, epilepsy

## Abstract

Various ketogenic diet (KD) therapies, including classic KD, medium chain triglyceride administration, low glycemic index treatment, and a modified Atkins diet, have been suggested as useful in patients affected by pharmacoresistant epilepsy. A common goal of these approaches is to achieve an adequate decrease in the plasma glucose level combined with ketogenesis, in order to mimic the metabolic state of fasting. Although several metabolic hypotheses have been advanced to explain the anticonvulsant effect of KDs, including changes in the plasma levels of ketone bodies, polyunsaturated fatty acids, and brain pH, direct modulation of neurotransmitter release, especially purinergic (i.e., adenosine) and γ-aminobutyric acidergic neurotransmission, was also postulated. Neuropeptides and peptide hormones are potent modulators of synaptic activity, and their levels are regulated by metabolic states. This is the case for neuroactive peptides such as neuropeptide Y, galanin, cholecystokinin, and peptide hormones such as leptin, adiponectin, and growth hormone-releasing peptides (GHRPs). In particular, the GHRP ghrelin and its related peptide des-acyl ghrelin are well-known controllers of energy homeostasis, food intake, and lipid metabolism. Notably, ghrelin has also been shown to regulate the neuronal excitability and epileptic activation of neuronal networks. Several lines of evidence suggest that GHRPs are upregulated in response to starvation and, particularly, in patients affected by anorexia and cachexia, all conditions in which also ketone bodies are upregulated. Moreover, starvation and anorexia nervosa are accompanied by changes in other peptide hormones such as adiponectin, which has received less attention. Adipocytokines such as adiponectin have also been involved in modulating epileptic activity. Thus, neuroactive peptides whose plasma levels and activity change in the presence of ketogenesis might be potential candidates for elucidating the neurohormonal mechanisms involved in the beneficial effects of KDs. In this review, we summarize the current evidence for altered regulation of the synthesis of neuropeptides and peripheral hormones in response to KDs, and we try to define a possible role for specific neuroactive peptides in mediating the antiepileptic properties of diet-induced ketogenesis.

The lack of a satisfactory response to pharmacological therapy in patients affected by epilepsy is a major neurological problem. In addition, in patients affected by epilepsy, antiepileptic drugs (AEDs) may induce serious side effects. Finding a solution to pharmacoresistance has been recently recognized as a priority by clinicians, parents, and stakeholders (1). Although various new AEDs are available to address refractory epilepsy, no improvements in treating drug resistance have been obtained, even with the release of additional new drugs (2). Alternatively to drug therapy, pharmacoresistant patients could be eligible for treatments such as: (i) neurosurgical resection of epileptic foci, (ii) intracranial or extracranial neurostimulation, and (iii) ketogenic diets (KDs).

Surgical resection appears to be effective in a high percentage (up to 73% are seizure free after 1 year post-surgery) of patients (3, 4). However, surgical intervention could also result in impairment of cognitive functions, such as verbal and visuospatial memory (5). In any case, the surgical approach to pharmacoresistance is a valuable alternative that is warranted when an epileptic focus is clearly identified, whereas in the opposite case alternative treatments, such as intracranial and extracranial neurostimulation and KDs, have to be considered.

Extracranial or intracranial stimulation has been proposed as a possible therapy for refractory epilepsy. In the first case, stimulation is applied to the vagal nerve (6). Recent meta-analyses of the results obtained with vagal stimulation show the limited efficacy of this approach (7, 8), limitations confirmed by a recent randomized active controlled trial in children (9). Other authors recommended the use of vagal nerve stimulation in children and adults, although the researchers concluded that more information about this technique is required to better define its impact on lifestyle and adverse effects (10). In addition to this extracranial neurostimulation technique, electrodes can be inserted directly into the brain to address specific cerebral regions, such as the cerebellum, centromedian thalamus, subthalamus, neostriatum, hippocampus, and brainstem. A few small trials have addressed the efficacy of intracranial stimulation procedures (11); however, these studies did not address the efficacy or safety of long term stimulation. Low- and high-frequency stimulation protocols have been evaluated, but the studies were inconclusive (12–14).

An effective approach for controlling pharmacoresistant seizures is represented by the nutritional induction of ketogenesis. Until now, the therapeutic use of KDs has been mainly limited to children (15), but recent studies suggest that KDs may be effectively used in adult patients affected by pharmacoresistant epilepsy as well (16). The clinical efficacy of KDs has been suggested by various investigations that were limited by the lack of randomization or a small patient sample (17). One study was based on the prospective analysis of a large (*n* = 150) sample of children, aged 1–16 years, with more than two refractory seizures/week, followed for 1 year (18). Interestingly, after 6 months of the KD, 71% remained on the diet, and 51% had a more than 50% decrease in seizure frequency (32% had more than 90% seizure reduction). Similar results were reported by a study that involved 20 children (3–16 years), who were treated for 30 months (19). Positive results were also reported in a controlled randomized study (20). However, limitations of the nutritional approach include adverse effects such as nausea, constipation, abdominal pain, or other less frequent complications (20–22). Low compliance with the dietetic regimen is also frequently present (22). Thus, in the long term effective control of seizures was achieved, at best, in approximately 25% of patients under KDs (21).

Dietetic treatments represent a less invasive approach for managing refractory epilepsy. Moreover, the possibility of understanding how KDs work could be of great help in discovering new treatments with possibly minimal side effects and the ability to control seizures unresponsive to currently available drug treatments. Further achievements may result from the discovery of new mechanisms involved in controlling ictogenesis, when studying the effects of KDs. Until now, the reason why ketogenesis is effective in restraining ictogenesis is undetermined. However, several possible mechanisms have been proposed to contribute to KD effects. This review is aimed at defining the current knowledge on the mechanisms putatively involved in the therapeutic effects of KDs. We also analyze the changes in endocrine axes related to fasting and ketogenesis, to tentatively identify the possible contribution of hormone peptides and neuropeptides to the antiepileptic effects of KDs.

## KDs: Common Characteristics and Several Differences

The classic KD is a low-carbohydrate/high-fat and normal protein diet that has been used as a treatment for pharmacoresistant epilepsy for a long time (22). The classic KD was initially proposed in 1921 (23) and thereafter was used extensively, although it was partially abandoned when AEDs became fully available. In spite of the development of new effective AEDs, the therapeutic application of the classic KD has survived over the decades as an alternative to drug therapy failure (24). During the course of the last 43 years, other modified KDs have been proposed, and the dietetic approach to epilepsy is being reevaluated as an effective alternative to control seizures refractory to at least two different AEDs. The most recently introduced KDs are all aimed at reducing the lipid content of the diet, in order to increase patient compliance and to limit the side effects (22, 25). Interestingly, a common characteristic of all KDs is maintaining a low-carbohydrate content whereas the fat content is presently less stringent (26).

Among the various types of KDs proposed to ameliorate the balance in diet macronutrients, the use of medium chain triglycerides (MCTs) as an alternative fat source was established at the beginning of the 1970s (27). The main constituents of the MCT diet are medium chain octanoic and decanoic fatty acids, which are absorbed more efficiently than long chain fatty acids and are readily transported to the liver by albumin. Conversely, long chain triglycerides (LCTs) are incorporated into chylomicrons and transported via the thoracic duct into the blood circulation. After hepatic uptake, MCTs are rapidly metabolized by liver mitochondria and, following oxidation, converted to ketone bodies. In contrast, LCTs require carnitine as a carrier to enter the mitochondrial biochemical machinery. These differences in metabolism facilitate more rapid and efficient oxidation of MCTs, supposed to result in a higher ketone yield per kilocalorie of dietary energy than that obtained from LCTs. Therefore, less total fat should be required to achieve the desired level of ketosis. With this method, more proteins and higher carbohydrate content may improve palatability and patient acceptance. However, a recent trial that compared the classic KD with the MCT diet demonstrated significantly higher induction of ketone bodies with the classic KD, but the clinical effectiveness of the two alternative diets was the same (21).

In the last few decades, two other types of KDs have been proposed with success. The modified Atkins diet (MAD) was introduced at the John Hopkins Hospital to restrict the fat content of the classic KD by allowing more carbohydrates and, especially, proteins, without limiting the total caloric intake (25). The MAD is based on the popular weight loss diet first described in 1972 (28), which was adapted to allow carbohydrate intake up to a maximum of 10–20 g/day, and by approximating a fat-to-carbohydrate/protein ratio of 1:1, thus less restrictive compared with the 3:1 or 4:1 ratios of the classic KD. Alternatively, the diet known as “low glycemic index treatment” (LGIT) was first described by Pfeifer and Thiele (29). This diet allows carbohydrate intake up to 40–60 g/day using a selection of carbohydrates with a low (<50) glycemic index. Consumption of carbohydrates with a low glycemic index minimizes the increase in glucose blood levels, keeping glycemia at a lower level after the meal. Food is not weighed but given in portion sizes. Protein, fat, and calorie intake under LGIT is considerably less strictly monitored compared with the classic KD. Unlike the MAD, a high-fat intake is not actively encouraged in LGIT protocols (Table 1).

**Table 1:** **Ketogenic diets (KDs) used to treat pharmacoresistant epilepsy are listed**.

Diet	Proponent(s)	Fasting	Ketogenic ratio	Hospitalization	Ketosis (plasma, mmol/L)	Urinary ketones	Fat (%)	Protein (%)	Carbohydrate (%)
Classic KD	Wilder (23)	Yes	2–4:1	Yes	2–5	3+ 4+	87–90	8	2–4
MCT	Huttenlocher et al. (27)	No	1.3–1.6:1	No	2.7–3.3	3+ 4+	70–75	10–15	15
MAD	Kossoff et al. (25)	No	0.9:1	No	Not specified	Variable	60–70	20–30	4–6
LGIT	Pfeifer and Thiele (29)	No	1:1	No	0.8–2	Variable	60–65	20–30	10–15

*Note that the ketogenic ratio (fats/carbohydrates–proteins) was progressively decreased by passing the classic KD to the others. Accordingly, a lower ketosis is allowed with diets alternative to classic KD. Fat, proteins, and carbohydrates are indicated in percentages of total calories*.

It is clear that varying the amount of carbohydrates in different diets induces different degrees of ketosis. This variability argues against a critical role of ketogenesis in the efficacy of KDs. A recent study by Neal and collaborators (21) compared the therapeutic effects of different levels of ketosis on seizures in a group of 125 children affected by intractable epilepsy. They were randomized to receive the classic KD or the MCT diet. Consistent with previous reports (30), this study showed that the mean acetoacetate level was significantly higher in the group maintained on the classic KD in the long term, even after 1 year of treatment, with no differences in the antiepileptic efficacy between the KD and MCT treatments. These data do not support the view of a causal relationship between ketosis and seizure control. Indeed, whereas some authors suggest that ketone bodies must be at a sufficient level to guarantee effective control of seizures (31), others disagree and support the view that a strict link between ketosis and seizure control may be questioned (32, 33). Interestingly, other findings support the hypothesis that a decrease in seizure frequency could depend on a fine balance between glucose reduction and ketone body production (34). Thus, ketosis could be just something more than an epiphenomenon of reduced glucose availability, and this latter could be the major player in controlling seizure activity, common to the different dietetic approaches.

## Mechanisms Thought to be Involved in KD Effects

During the past few decades, interest in understanding the key mechanism of KDs has steadily been grown. Apparently, the mechanisms of KDs could be easily delineated. This alternative therapeutic approach was designed to mimic the biochemical changes associated with fasting, since fasting was proven to be effective in reducing seizure frequency (22, 23, 25). Thus, the reproduction by dietary manipulation of the ketotic state associated with fasting appears to be the way by which pharmacoresistant epilepsy can be controlled. Ketosis is characterized by increased levels of ketone bodies (β-hydroxybutyric acid, acetoacetic acid, and acetone) that can be achieved by reducing blood glucose levels (35–37). Ketone bodies are the obvious candidates to investigate the mechanism of action by which KDs may control seizure activity. According to Gilbert et al. (31), serum β-hydroxybutyrate levels may correlate with seizure reduction. Although this observation was confirmed by others (38), various studies addressing the anticonvulsant activity of β-hydroxybutyrate failed to demonstrate any relevant effect of this ketone body in different animal models (37, 39–41) or even *in vitro* (42). However, some investigators demonstrated an anticonvulsant effect of acetoacetate and of its metabolite acetone in various seizure paradigms, including the maximal electroshock test, the subcutaneous pentylenetetrazole test, the amygdala kindling test, and atypical absence seizures, which characterize the Lennox–Gastaut syndrome (43, 44). Interestingly, acetone may act by opening the two-pore-domain potassium channels (K2p), whose characteristic is to hyperpolarize neurons thus limiting neuronal excitability (45). In spite of these encouraging results, *in vivo* experimental (46) and clinical studies (47) showed that acetone levels did not rise sufficiently to suggest the involvement of ketone bodies in the therapeutic effects of KDs. Specifically, according to proton magnetic resonance spectroscopy studies, acetone levels were around 0.7 mM in the brain of patients maintained on a KD (47) or, based on measurements in the cerebrospinal fluid (48), even lower. In addition, the acetone plasma levels effective in rats must be higher than 2 mM; generally, such levels were never obtained under different KD treatments (46). These findings raise several questions: although investigations of animal models remain the required approach for studying the antiepileptic effects of KDs (49, 50), in every tested animal model the efficacy of KDs has strongly depended on the specific seizure model. Moreover, according to some authors, KDs are not effective in seizure models as they are in patients, with the exception of the 6-Hz corneal stimulation paradigm (51, 52) (Table 2).

**Table 2:** **Effects of the ketogenic diet (KD) in seizure and epilepsy models**.

Species	Seizure induction	KD’s effects	Reference
Mice	Bicuculline injection	Reduced seizures	(53)
Rat	Audiogenic seizures	Decreased latency to seizure onset	(54)
Rat	Amygdala kindling	Protection against the focal onset of kindled seizures but not on seizure spread	(55)
Rat	KA injection	Decreased supragranular mossy fiber sprouting	(56)
Juvenile mice	Flurothyl test	Protection against mortality	(57)
Rat	MES	More severe seizures	(58)
Rat	PTZ injection	Protection against seizures	(37, 58)
Juvenile mice	EL, human idiopathic epilepsy	No effects	(50, 59)
Rat pups	PTZ infusion test	Improved survival	(60)
	ECS	Small threshold elevation	
	Maximal PTZ test	No effects	
	Subcutaneous PTZ test	No effects	
Juvenile mice	6-Hz test (corneal)	Marked elevation of the seizure threshold	(51, 52)
Mice	Succinic semialdehyde dehydrogenase/γ-aminobutyric acid deficiency	Increased lifespan	(61)
		Decreased ataxia	
		Decreased weight loss	
		Decreased other abnormalities	

*Notably, the diet is paradoxically proconvulsant in the maximal electroshock stimulation (MES) test, weakly active respectively in the pentylenetetrazole (PTZ) test, the kainic acid (KA) model, the electroconvulsive shock (ECS) paradigm, and the flurothyl test, but KD is highly active in the 6-Hz model*.

Inconsistencies between the therapeutic levels of ketone bodies and doses required to produce anticonvulsant activity in *in vivo* and *in vitro* models are at the basis of the absence of clear explanations of KD effects. Although evidence of the direct involvement of ketone bodies in KD effects is lacking, changes in the functioning of different neurotransmitters have been described in the presence of ketosis and were extensively analyzed in a recent review (37). Relevant changes in γ-aminobutyric acid (GABA) and glycine levels were demonstrated in the cerebrospinal fluid of patients maintained on KDs, suggesting the enhanced activity of inhibitory neurons (62, 63). Conversely, animal studies failed to demonstrate any change in GABA levels measured in the brain homogenates of rodents maintained on a KD, disconfirming a role for GABA in mediating KD effects (64). Although the overall evidence is against upregulation of GABA by KDs in animal models, electrophysiological recordings demonstrated that network excitability is diminished in animals fed with KDs (65). Another hypothesis is based on the depression of glutamatergic transmission (66), since acetoacetate and, less efficiently, β-hydroxybutyrate can compete with chloride, which is an allosteric activator of vesicular glutamate transport, and this competition could lead to decreased glutamate release. However, the required concentrations of ketone bodies to reduce glutamate release appear to be far from those found in patients (37).

A more recent attempt to identify a mechanism for KDs suggested the involvement of K_ATP_ channels, hyperpolarizing ion channels that are opened by low energy levels, i.e., decreased ATP. It has been argued that ketone bodies, which directly enter the Krebs cycle, could reduce glycolytic ATP production thus activating K_ATP_ channels (67). Data obtained in *in vitro* (68, 69) and *in vivo* (70) experiments supported the involvement of K_ATP_ channels. However, the evidence that ATP levels are decreased by KDs is lacking (71–73).

Several other mechanisms have been taken into account, including: (i) cerebral acidosis by itself, tentatively reproduced by the administration of inorganic acids (74). However, animal studies have shown that initial acidosis is then compensated and brain pH results are actually normal (75). Another mechanism is (ii) changes in the accumulation of lipids, such as polyunsaturated fatty acids (PUFAs) (76). PUFAs activate the peroxisome proliferator-activated receptor-α and regulate the transcription of genes that enhance energy metabolism (77). The third mechanism is (iii) increased ATP synthesis leading to the accumulation of adenosine (78). Adenosine stimulates adenosine A1 receptors and may reduce spontaneous seizures, as shown in mice with elevated levels of adenosine (79). The fourth mechanism is (iv) the noradrenergic tone: noradrenaline may modulate the propensity to develop seizures (80, 81), and KDs were shown to enhance noradrenaline tissue levels. Mice lacking the gene for dopamine β-hydroxylase failed to exhibit an elevated flurothyl threshold when fed a KD (82). An intriguing consequence of KD effects on noradrenergic signaling could be the stimulation of the release of food intake modulators, which are known to work in conjunction with hypothalamic neurotransmitters.

## New Hypothesis: Peptide Hormones as Possible Mediators of KD Effects

Several peripheral peptides produced in the gut and associated tissues have been suggested to link changes in body metabolism with central nervous system functions and, thus, may be critical regulators in various pathophysiological conditions, including control of neuronal excitability in epilepsy (83–86). Peptide hormones are short molecules composed of approximately 3–100 amino acid residues, characterized by a structure simpler that than for proteins. Some are also synthesized by neurons and termed neuropeptides. Peptides and neuropeptides act through G-protein coupled receptors diffusely expressed in the nervous system. Notably, neuropeptides can act at a distance by diffusing from the releasing site in the extracellular space to interact with extrasynaptic receptors and produce long-lasting effects (87–89). At least one receptor for each peptide hormone has been identified, which means that presumably several hundred receptors can affect a multitude of intracellular transduction pathways, complicating the interpretation of their functions. Many peptides are expressed in neurons that co-express at least one classic transmitter and often more than one neuropeptide (90, 91). Physiologically, peptides mainly behave as neuromodulators with prolonged action on multiple physiological and behavioral actions. The long term effects of peptides depend on the modulation of gene expression via activation of a particular cascade of intracellular signaling molecules (92, 93). However, rapid effects of peptides on neuronal activity by interactions among peptide receptors and ion channels or exchangers have also been described (94, 95). The versatility of peptide hormones and neuropeptide actions explains their multiple physiological effects. In line with the localization of neuropeptides in the hippocampus, amygdala, hypothalamus, striatum, and spinal cord, physiological functions include emotional reactivity, learning and memory, neuroendocrine response to stress, hippocampal synaptic plasticity, and hypothalamic neurotransmission (90, 96–98).

### Peptide hormones involved in the control of metabolism

Metabolism is regulated by various neural and hormonal systems. The latter include hormone peptides that display anorexigenic or orexigenic properties. Peripheral anorexigenic hormones are produced in different tissues, spanning the gut to the pancreas and, finally, including the adipose tissue. Appetite-reducing peptides released by the gut include cholecystokinin (CCK) (99, 100), glucagon-like peptide-1 (GLP-1) (101, 102), peptide YY (PYY) (103, 104), and oxyntomodulin (105, 106). Anorexigenic peptides from the pancreas are the pancreatic polypeptide (PP) (107, 108), glucagon (109), insulin (110, 111), and amylin (112). Finally, hormones that suppress appetite synthesized in the adipose tissue are leptin (113, 114), adiponectin (115), and resistin (116). In contrast to this variety of appetite-suppressing hormones, ghrelin is the only peripheral peptide with orexigenic properties and is mainly produced in the stomach (117, 118). Interestingly, some of the hormones displaying food intake regulatory properties also possess antiseizure effects (86, 119) (Table 3).

**Table 3:** **Changes in different neuroactive peptide levels in animals and humans during various metabolic states, including anorexia, prolonged fasting, or a ketogenic diet (KD)**.

Peptides	Rodents	Reference	Human	Reference
Leptin	↓KD, fasting	(120, 121)	↑ ↓Anorexia, fasting	(122–124)
Insulin	↓KD	(125–127)	↓KD, anorexia	(124, 128)
Adiponectin	↑Anorexia, fasting	(129, 130)	↑ ↓Anorexia	(131, 132)
NPY	↑KD, fasting	(133, 134)	↓Anorexia	(122, 135)
Galanin	↑KD, fasting	(136)	↑Anorexia	(137)
Ghrelin	↑KD, anorexia	(138, 139)	↑Anorexia, fasting	(140, 141)
IGF-1	↑KD	(142)	↓Anorexia	(143)
CCK	↓Anorexia, KD	(144, 145)	↑Anorexia	(146, 147)

*Most studies demonstrated increased adiponectin, neuropeptide Y (NPY), galanin, or ghrelin levels or decreased leptin and insulin levels, in animals and patients exposed to similar conditions. Other abbreviations: CCK, cholecystokinin; IGF-1, insulin-like growth factor-1*.

The regulation of energy balance depends on the coordination of multiple peripheral and central systems and is tightly modulated by neuropeptides (148, 149). The critical regulation of food intake is thought to occur in the hypothalamus. Neurons in the parvocellular part of the paraventricular hypothalamic nucleus produce the anorectic neuropeptide corticotropin releasing factor (CRF) (150), while those in the lateral hypothalamus produce orexigenic neuropeptides such as orexin and melanin-concentrating hormone (151). The arcuate nucleus of the hypothalamus contains two distinct neuronal populations that produce orexigenic and anorexigenic peptides, which seem to play antagonistic roles in energy balance control and that are regulated by leptin and insulin (152–154). The “anorexigenic” population is located in the lateral subdivision of the arcuate nucleus and produces the anorectic neuropeptides proopiomelanocortin and cocaine- and amphetamine-regulated transcript (155, 156). The “orexigenic” population of neurons is located in the medial subregion of the arcuate nucleus and produces orexigenic neuropeptides such as agouti-related peptide and neuropeptide Y (NPY) (30, 157). The hypothalamic cell groups are specially tuned to sense metabolic changes, to regulate hormonal secretion, energy homeostasis, and metabolism. The mechanisms for controlling food intake during fasting or a KD involve a complex interplay between the peripheral systems controlling gastrointestinal peptide secretion and the central nervous system. These neuronal systems include neuropeptides and peripheral neuroactive peptides such as CRF, opioids, NPY, CCK, orexin, galanin, and leptin, as well as monoamines (serotonin, dopamine, and noradrenaline).

The complex machinery involved in regulating food intake in relation to body metabolism is also strictly linked to the characteristics of the meal. For instance, ghrelin stimulates sugar intake (158), whereas the neuropeptide oxytocin inhibits carbohydrate but not fat intake (159). Galanin, enkephalins (160), and β-endorphin (161) promote the preference for fat meals. Conversely, macronutrients can influence peptide hormone release. This is the case for lipids, which exert a satiating effect that is probably related to the increased secretion of GLP-1, PYY, and CCK (162). Interestingly, in the presence of increased ghrelin levels a high-fat diet can prevent any change in food intake due to hormonal regulation (163). Thus, the classic KD could deeply alter peptide hormone levels and their function by virtue of the prevalent lipid content of the diet. This effect could be further influenced by the presence of ketone bodies or, in some cases, by weight loss. For instance, in the case of weight loss caused by a KD, insulin and leptin are both reduced when compared to pre-diet levels (164). Ghrelin is not affected by weight loss induced by a KD (164), but is increased in the case of anorexia (165, 166) or cachexia (167, 168). Upregulation of ghrelin levels during the course of starvation is expected, since ghrelin production is stimulated by a negative energy balance as a compensatory response.

### Peptide hormones that modulate seizures

Peptide hormones and neuropeptides have been extensively investigated in the context of animal models of epilepsy and with clinical studies (Table 4). Lines of evidence point to an important role for peptide hormones and neuropeptides in regulating neuronal activity during seizures. In particular, peptide hormones such as adiponectin, ghrelin, and leptin and neuropeptides such as CCK, galanin, NPY, and somatostatin appear to be promising candidates for future research on new treatments for epilepsy.

**Table 4:** **Changes in the levels of neuroactive peptides following seizures**.

Peptide	Animal model of epilepsy	Change	Reference
NPY	Electrical kindling	↑	(169)
	KA model	
Galanin	KA model	↓	(170)
	PTZ model	
Ghrelin	PTZ model	↓	(171, 172)
CCK	KA model	↓	(173, 174)
	Pilocarpine model	
Leptin	Penicillin model	↑	(172, 175)
	KA model	
Adiponectin	KA model	↓	(176, 177)

*Note that galanin, ghrelin, cholecystokinin (CCK), and adiponectin decreased, whereas neuropeptide Y (NPY) and leptin levels increased in response to different convulsive stimuli. Other abbreviations: KA, kainic acid; PTZ, pentylenetetrazole*.

Adiponectin is produced by adipocytes to regulate fat oxidation and sensitivity to insulin. Adiponectin plasma levels decreased in metabolic syndrome, suggesting the involvement of this hormone in the insulin resistance and hyperlipidemia characterizing the syndrome (178). Adiponectin knockout (KO) mice developed vascular injury and inflammation when exposed to a high-fat diet. Conversely, adiponectin protected from cerebral ischemia due to modulatory properties on inflammation (179, 180). Beneficial effects were also demonstrated in the kainate model of temporal lobe epilepsy, in which adiponectin reduced neuronal cell loss (176). Lee et al. (177) investigated the effects of kainate administration in adiponectin KO mice fed a high-fat diet, compared with the wild-type (WT) controls, by monitoring the blood parameters related to altered glucose and lipid metabolism, the epileptic response to kainate and acute tissue damage. Although the authors were not able to document the effects of kainate with electroencephalography, the seizure score was always lower in the WT mice compared with the KO mice, which also were characterized by metabolic changes such as glucose intolerance. In another experiment, kainate was directly injected in the hippocampus, and the damage was examined after 2 weeks. Glial reaction was more intense in the adiponectin KO mice compared with the WT mice, but the neuronal cell loss and granule cell dispersion were also significantly more pronounced in the investigated model of metabolic syndrome. These last findings suggest that a high-fat diet causes hippocampal damage in the long term. To exclude the role of adiponectin deletion in the observed proconvulsive effects of the diet, the authors replicated the same experiments with kainate injection in KO mice fed a standard diet, and observed a response similar to that found in the WT mice. In addition, the authors demonstrated a strong correlation between glucose intolerance, cholesterol, fat mass, free fatty acids, and seizure severity. In contrast, hypertriglyceridemia and body weight were not correlated with epileptic activity (177).

Ghrelin has been suggested to act as an anticonvulsant in various seizure models. In particular, ghrelin was found to delay the onset of myoclonic jerks and tonic generalized seizures induced by pentylenetetrazole (181). However, in the same experiment ghrelin increased the duration of generalized clonic seizures. In the penicillin-induced neocortical seizure model, ghrelin decreased the frequency of interictal spiking activity (182). Furthermore, in the kainate model of *status epilepticus* (SE), this hormone significantly attenuated seizures and SE duration, resulting in decreased cell loss (183). Additional experiments based on the pilocarpine model confirmed the anticonvulsant effects of ghrelin (119), but this activity was not strong enough to prevent the development of SE (184, 185). Intriguingly, better findings were instead obtained by using ghrelin analogs such as des-acyl ghrelin and EP-80317 (184). Conversely, the ghrelin full agonist JMV-1843 failed to prevent pilocarpine-induced seizures (184, 185). This picture was further complicated by the discovery that KO mice for the ghrelin receptor were resistant to pilocarpine-induced seizures (119). These findings led to the proposal that the growth hormone secretagogue receptor 1a (GHS-R1a), which is endowed with high intrinsic activity, was reduced on the cell surface as a consequence of interaction with ghrelin, and that this phenomenon was probably the explanation for the anticonvulsant effects of ghrelin (119). However, ghrelin is rapidly converted to des-acyl ghrelin, and this metabolite could be responsible for the effects attributed to ghrelin administration (186). Interestingly, des-acyl ghrelin could also modulate other targets through binding to the CD36 scavenger receptor, which is involved in regulating food intake and in signaling cascades that lead to neuronal dysfunction and initiation of cell death (187).

Leptin’s anticonvulsant activity was originally shown in two different models: Xu et al. (188) showed that leptin administered by the intranasal route delayed the effects of the convulsant pentylenetetrazole, as well as reduced seizures evoked by 4-aminopyridine. In addition, the same authors confirmed the anticonvulsant effects of leptin in an *in vitro* model and suggested that this effect was related to the depression of glutamatergic neurotransmission (188). In agreement with these data, pentylenetetrazole-induced seizures were more severe in leptin KO (ob/ob) mice (189). However, at odds with previous findings, other experiments showed that leptin potentiated neocortical electrographic seizures elicited by penicillin, probably by interfering with the production of nitric oxide (190). Leptin’s proconvulsant activity was also suggested by another study (191), in which leptin pretreatment increased the number of animals exhibiting convulsions in response to *N*-methyl-d-aspartic acid or kainate administration. Recently, the possible involvement of the cannabinoid CB1 receptor in leptin proconvulsant activity in the penicillin model has also been suggested (175). These discrepancies are still unexplained and could depend on the seizure model. In particular, leptin appears to play a modulatory role rather than to be an anticonvulsant.

The expression of neuropeptides changes dramatically in animal models of epilepsy, especially in the hippocampal formation (Table 4). The implication of these peptides in modulating seizures is likely due to their ability to decrease excitability in the brain. Moreover, their brain concentration changes after spontaneous seizures, and this may contribute to enhanced susceptibility to seizures (169). Importantly, recurrent spontaneous seizures were suppressed by overexpression of NPY in the hippocampus using adeno-associated virus vectors (192). Several studies have investigated the therapeutic potential of these neuropeptides to suppress seizure activity in animals, in various epilepsy models (193). These studies were in agreement with data on NPY or galanin KO mice, which presented an increased propensity to develop seizures (170, 194). Another interesting aspect of neuropeptides is their neuroprotective role after brain injury, a condition that can lead to the development of chronic epilepsy. In particular, NPY promotes neurogenesis in the subventricular zone and the subgranular zone of the adult mouse brain (195, 196). These aspects and others were the focus of recent, extensive reviews about the role of neuropeptides and their receptors (86, 197).

### Could peptide hormones be mediators of KD antiepileptic effects?

Patients affected by epilepsy and treated with AEDs present with various changes in the plasma levels of hormones related to metabolism, by which patients could also develop metabolic disorders. In a pioneering study of 40 patients treated with valproate, obesity was found in 15 epileptic patients who also displayed increased leptin and insulin levels and decreased ghrelin and adiponectin levels, when compared with subjects with normal body weight (198). In contrast, another investigation suggested ghrelin production increased (+70%) in patients affected by epilepsy (199). These observations were confirmed by examining the effects of valproic acid treatment on ghrelin in a sample of children characterized by increased body weight, body mass index, and height, in which serum ghrelin levels were higher than in controls and negatively correlated with insulin-like growth factor-1 (IGF-1) and insulin-like growth factor binding protein-3 (200). Further studies on ghrelin in patients suffering from epilepsy suggested that ghrelin levels are lower in adults treated with various AEDs (201), as well as in children treated with carbamazepine or valproic acid (202). Finally, a relationship between peptide hormones and seizures has been suggested by a study in which prolactin, nesfatin-1, and ghrelin were evaluated within 5 min after a convulsion: the serum levels of prolactin and nesfatin-1 increased, and interestingly, the ghrelin levels were abated for at least 24 h (172). Since ghrelin is a promising candidate in view of its modulatory properties in seizures (119, 203), the changes in this hormone regulation found in patients with epilepsy are of major interest, whereas other changes in peptide hormone regulation, involving leptin, galanin, and NPY (204, 205), must be clearly defined.

The metabolism of peptide hormones such as ghrelin and leptin is modified by seizures and AEDs, suggesting that they could also be affected by KDs. In spite of the important role peptide hormones have in regulating body metabolism (206–208), only a few studies have examined the changes in peptide hormone levels in humans treated with KDs (164, 209, 210). Fraser et al. (209) analyzed the acute anti-inflammatory effects of a KD in patients affected by rheumatoid arthritis and found significant decreases in serum leptin and IGF-1, similar to those observed during acute starvation. These results were probably explained by the weight loss induced by the diet. Significant changes in various peptide hormones associated with weight loss in obese subjects maintained on a KD were also described (164). More interesting for epilepsy and KD treatment, decreased fasting insulin and leptin levels were reported in children affected by Glut-1 deficiency syndrome and treated with a 3:1 KD, whereas adiponectin levels were unmodified (210). In this last study, the observed changes in insulin and leptin levels were not dependent on weight loss. More data have been obtained by studying animal models: Kinzig and collaborators (138) found that rats maintained on a low-carbohydrate/high-fat diet had increased adiposity when compared with controls, accompanied by increased leptin and ghrelin levels and decreased insulin plasma levels. Thus, insulin, but not leptin, was downregulated as in children affected by Glut-1 deficiency syndrome and maintained on KD therapy (210). The high-fat content of the diet seems to be an important requisite to stimulate leptin production, since the leptin levels of rats maintained on a low-carbohydrate/high-protein diet did not vary (211). More interestingly, ghrelin induces ketone bodies production when administered to humans (212), and ghrelin upregulation by a low-carbohydrate/high-fat diet, as demonstrated in rats (138), could be a critical phenomenon in mediating KD effects.

Ghrelin is an important regulator of energy balance that is released from the stomach in response to fasting (180). In addition to regulating appetite, ghrelin stimulates the release of growth hormone (GH) through the GHS-R1a. Other different ghrelin-related peptides are produced in the stomach, including des-acyl ghrelin, which is also obtained by ghrelin desacylation in the periphery. According to some authors, des-acyl ghrelin represents approximately 90% of total circulating ghrelin-related peptides (213). It regulates the energy balance independently of ghrelin. Indeed des-acyl ghrelin is unable to activate GHS-R1a; thus, this peptide is devoid of GH-releasing properties. Interestingly, fasting alters the secretion of ghrelin and des-acyl ghrelin, but des-acyl ghrelin appears to be a long-lasting signal associated with a negative energy balance. Indeed, when fasting is prolonged and implies a negative energy balance, such as in patients affected by anorexia or cachexia, des-acyl ghrelin is more stably elevated than ghrelin, and the des-acyl ghrelin/ghrelin ratio is also significantly increased (214). Prolonged fasting is mimicked by KD in patients affected by epilepsy. These data suggest that ghrelin-related peptides, especially des-acyl ghrelin, could be stably increased in patients affected by pharmacoresistant epilepsy and receiving a KD treatment. Ghrelin, but not des-acyl ghrelin, modulated the release of GABA via the GHS-R1a receptor (215) and increased the GABAergic tone. This phenomenon probably explains the counteraction of pentylenetetrazole and penicillin proconvulsive activities observed after ghrelin administration (181, 182). However, des-acyl ghrelin, which does not activate GHS-R1a, also displays anticonvulsive properties by unknown mechanisms, as shown in models of pharmacologically induced acute seizure (184). Experiments based on administration of EP-80317, one of the various molecules developed by modifying ghrelin, confirmed the existence of anticonvulsive effects independent of GHS-R1a receptor activation (203). Des-acyl ghrelin was previously considered an inactive ghrelin metabolite. Although des-acyl ghrelin lacks most of the biological properties of ghrelin, des-acyl ghrelin is now recognized as a hormone because it can modulate food intake by a mechanism independent of GHS-R1a stimulation, depending on circadian rhythmicity and on fasting pre-exposure (216, 217). In addition, des-acyl ghrelin depresses gastrointestinal motility independently of ghrelin, by a central mechanism involving the CRF type 2 receptor (218). At the cellular level, des-acyl ghrelin increased medium chain fatty acid uptake in cardiomiocytes, whereas ghrelin was ineffective; conversely, ghrelin inhibited the increase in glucose uptake normally induced by insulin, but des-acyl ghrelin did not. Thus, des-acyl ghrelin appears to regulate the body metabolism in a way different from ghrelin, without causing dramatic changes in food intake or body development. This hypothesis is confirmed by studies on des-acyl ghrelin transgenic mice that showed a limited decrease in linear growth and slightly reduced fat mass, phenomena related to the negative energy balance produced by des-acyl ghrelin (219). These properties make des-acyl ghrelin interesting from a pharmacological point of view, also for the nervous system. Pharmacokinetic experiments showed that des-acyl ghrelin enters the brain by non-saturable transmembrane diffusion and is sequestered once within the central nervous system to exert its activity there (218). Thus, the demonstration that des-acyl ghrelin could mediate the anticonvulsive effects of KD could pose the basis for a pharmacological exploitation of this hormone and its possible analogs in epileptic disorders.

Different ghrelin analogs have been developed by simply modifying the peptidergic structure of this hormone, obtaining GH secretagogues with diverse pharmacological profiles (203, 220, 221). Some molecules have been developed to be orally administered and, in general, present an advantageous pharmacokinetic profile when compared with ghrelin. In particular, JMV-1843 is a potent GHS-R1a agonist that stimulates GH release and food intake, whereas JMV-2959 appears to be an antagonist (221, 222). Hexarelin presents less marked GH stimulating properties, which are completely lost with molecules functionally closer to des-acyl ghrelin, such as EP-80317 (187). Interestingly, EP-80317 interacts with CD36 (223), a type B scavenger receptor involved in internalizing oxidized low-density lipoprotein, which initiates a signaling cascade that regulates microglial recruitment and activation, and subsequently, secretion of inflammatory mediators such as interleukin (IL)-1β and IL-6. These mediators, particularly IL-1β, were shown to promote neuronal overexcitation and seizures (224). Indeed, Bulgarelli et al. (187) demonstrated that EP-80317, hexarelin, and des-acyl ghrelin in the nanomolar range effectively counteract the stimulation of IL-1β and IL-6 synthesis in microglial cells lacking the specific ghrelin receptor GHS-R1a, suggesting the involvement of the CD36 receptor in these effects. However, the expression of this receptor in the brain is limited to a few regions (225), and thus, des-acyl ghrelin anticonvulsive effects, whether mediated through CD36 activation, have to be restricted mainly to microglial cells. Thus, new molecular pathways, possibly important for pharmacoresistant epilepsy, could be implicated in des-acyl ghrelin anticonvulsive effects, but these relationships remain to be investigated in appropriate animal models to elucidate their possible involvement in KD beneficial effects.

## Conclusion

Neuroactive peptides involved in the control of metabolism are putative candidates as mediators of KD effects in pharmacoresistant epilepsy (119, 184). Fasting is mimicked by KDs through inducing ketone bodies and by lowering glucose plasma levels, effects that depend on the activation of some endocrine axis and on the suppression of others. The release of peptides such as insulin, leptin, ghrelin, des-acyl ghrelin, and adiponectin is closely related to food intake or fasting. These hormones have also been related to seizure induction (insulin) or suppression (leptin, ghrelin, des-acyl ghrelin, and adiponectin). Thus, several peripherally originated neuroactive peptides could play a role in modulating epileptic activity in the brain, and their changes in response to fasting or KDs are of primary interest in the attempt to clarify which mechanism could be responsible for the efficacy of dietetic approaches to pharmacoresistant epilepsy. A promising hormonal product is represented by des-acyl ghrelin. This neuroactive peptide is increased in catabolic states and was shown to exert anticonvulsant activity in models of SE (184). A des-acyl ghrelin analog, EP-80317, was also effective in preventing seizure induction by pilocarpine when preventively administered in rats (203). In future studies, we suggest that des-acyl ghrelin may deserve thorough investigation as a possible mediator of KD effects.

Identifying KD mediators could represent a significant advancement in treating pharmacoresistant epilepsy. Although patients suffering from pharmacoresistance could take advantage of therapeutic alternatives such as surgical resection of epileptic foci, intracranial or extracranial neural stimulation, or KDs, compliance with these approaches is not optimal. Many reasons could discourage a patient from adopting any of these therapeutic alternatives to drug treatment: (i) the need for invasive surgery; (ii) possible impairment of cognitive and memory functions caused by intracranial or extracranial stimulation; and (iii) gastrointestinal complications related to KDs such as nausea, constipation, and abdominal pain. Clarification of the neuronal and molecular mechanisms of antiepileptic effects of KDs will propose a specific therapeutic solution for pharmacoresistant epilepsy.

## Conflict of Interest Statement

The authors declare that the research was conducted in the absence of any commercial or financial relationships that could be construed as a potential conflict of interest.
